# SalMotifDB: a tool for analyzing putative transcription factor binding sites in salmonid genomes

**DOI:** 10.1186/s12864-019-6051-0

**Published:** 2019-09-02

**Authors:** Teshome Dagne Mulugeta, Torfinn Nome, Thu-Hien To, Manu Kumar Gundappa, Daniel J. Macqueen, Dag Inge Våge, Simen Rød Sandve, Torgeir R. Hvidsten

**Affiliations:** 1 Centre for Integrative Genetics (CIGENE), Department of Animal and Aquacultural Sciences, Faculty of Biosciences, Norwegian University of Life Sciences, Ås, Norway; 2 The Roslin Institute and Royal (Dick) School of Veterinary Studies, The University of Edinburgh, Midlothian, UK; 3 Faculty of Chemistry, Biotechnology and Food Science, Norwegian University of Life Sciences, Ås, Norway

**Keywords:** Transcription factor binding sites, Regulatory networks, Salmonid genomics, Gene regulation, Web tool

## Abstract

**Background:**

Recently developed genome resources in Salmonid fish provides tools for studying the genomics underlying a wide range of properties including life history trait variation in the wild, economically important traits in aquaculture and the evolutionary consequences of whole genome duplications. Although genome assemblies now exist for a number of salmonid species, the lack of regulatory annotations are holding back our mechanistic understanding of how genetic variation in non-coding regulatory regions affect gene expression and the downstream phenotypic effects.

**Results:**

We present SalMotifDB, a database and associated web and R interface for the analysis of transcription factors (TFs) and their *cis*-regulatory binding sites in five salmonid genomes. SalMotifDB integrates TF-binding site information for 3072 non-redundant DNA patterns (motifs) assembled from a large number of metazoan motif databases. Through motif matching and TF prediction, we have used these multi-species databases to construct putative regulatory networks in salmonid species. The utility of SalMotifDB is demonstrated by showing that key lipid metabolism regulators are predicted to regulate a set of genes affected by different lipid and fatty acid content in the feed, and by showing that our motif database explains a significant proportion of gene expression divergence in gene duplicates originating from the salmonid specific whole genome duplication.

**Conclusions:**

SalMotifDB is an effective tool for analyzing transcription factors, their binding sites and the resulting gene regulatory networks in salmonid species, and will be an important tool for gaining a better mechanistic understanding of gene regulation and the associated phenotypes in salmonids. SalMotifDB is available at https://salmobase.org/apps/SalMotifDB.

**Electronic supplementary material:**

The online version of this article (10.1186/s12864-019-6051-0) contains supplementary material, which is available to authorized users.

## Background

Salmonid fish are widely studied due to their ecological importance, unique evolutionary history, and large economic impact as an aquaculture species [1]. Recently, the availability of genomic resources for salmonids has exploded, with chromosome level assemblies for several species including Atlantic salmon [2], Arctic charr [3], Chinook salmon [4] and Rainbow trout [5], and draft assemblies for other species including Coho salmon (GCF_002021735.1).

These new resources have sparked a number of studies to understand the genetic basis for life history trait variation in the wild [6–8], identify genes and genetic variation associated with economically important aquaculture traits [9–12], and shed light on consequences of the salmonid-specific whole genome duplication on gene regulatory evolution [2, 3, 13, 14]. However, reaching a mechanistic understanding of how regulatory DNA changes effect trait variation and give rise to novel genome regulation still remains a major challenge.

Regulatory DNA plays an important role in trait variation within populations [15] and also for evolution of novel traits at the evolutionary time scale [16]. Most causal variants underlying phenotypic variation in vertebrates are non-coding variants in regulatory regions [15]. Such variants likely alter transcription factor binding propensity with consequences for gene regulation [17]. Gene regulatory evolution is partly shaped by the birth and death of *cis*-regulatory elements [18], which in many cases are linked to transposable element insertions [19]. Despite the great genomic resources now available for salmonids, the gene regulatory landscape is poorly characterised and no resource so far exists for predicted *cis*-regulatory elements. This is a hindrance for further progress in understanding the mechanistic basis of salmonid traits and adaptations.

In this paper, we describe a new database containing predicted transcription factor binding sites in salmonid genomes, called SalMotifDB. The database is accessible through salmobase (http://www.salmobase.org/) [20], and can be queried both through a graphical user interface and an R package. It includes tools to extract gene regulatory network information, as well as tools to perform tests for overrepresented TF-binding sites in the *cis*-regulatory regions of user specified genes.

## Construction and content

### The motifs

**Fig. 1:**
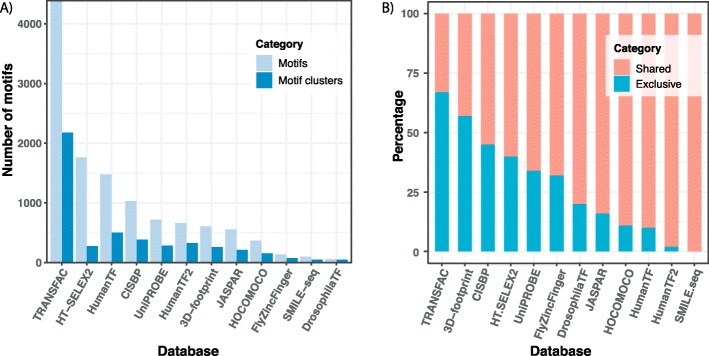
Motif clustering. **a** The light blue bars represent the number of motifs from a particular database while the dark blue bars indicate the number of motif clusters from that same database. Each cluster contains similar motifs and is represented by one non-redundant central motif. **b** The percentage of central motifs representing clusters that are specific to one database (Exclusive) and representing clusters containing motifs from more than one database (Shared)

A total of 19,845 metazoan transcription factors (TF) and their DNA binding sites were obtained from CISBP [21], JASPAR [22], 3D-footprint [23], UniPROBE [24], HumanTF [25], HumanTF2 [26], HT-SELEX2 [27], SMILE-seq [28], FlyZincFinger [29], HOCOMOCO [30], DrosophilaTF [31] through footprintDB [29] and TRANSFAC© [32]. Binding sites are represented as Position Specific Scoring Matrices (PSSMs), henceforth referred to as motifs. To mitigate motif redundancy, we employed the standard approach of RSAT (Regulatory Sequence Analysis Tool) [33]. Specifically, we used the RSAT *matrix-clustering* tool, with parameters *-quick*, *−cor = 0.65* and *-Ncor = 0.8*. We first clustered motifs within each database (Fig. 1a) and then clustered the central motif of these database-specific clusters across databases (Additional file 1: Figure S1). Here, the central motif of a cluster is the motif with the highest similarity to other motifs in that cluster, as calculated by *matrix-clustering*. The final clustering resulted in 3092 motif clusters, and while all the original motifs are retained in SalMotifDB, the motif clusters and their representative (central) motifs are used to organize and remove redundancy in results throughout our tools. Different databases contributed with widely different numbers of motifs (Fig. 1)a, as well as with different numbers of exclusive motifs not found in other databases (Fig. 1b). TRANSFAC was clearly the dominating database in both respects.

**Fig. 2:**
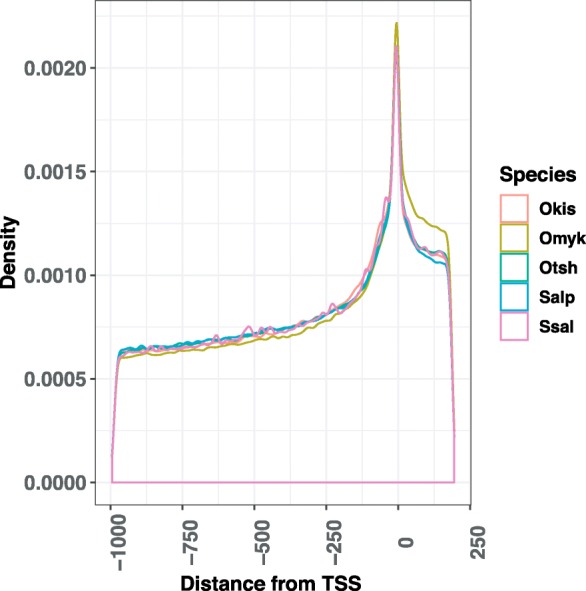
Positional bias of motif matches. Distribution of motif matches in promoters from 1000 bps upstream of transcription start site to 200 bps downstream - for each salmonid species

FIMO (Finding Individual Motif Occurrence) [34] is a widely used tool for mapping known motifs to genomes and has performed well in comparative studies [35]. We used FIMO to obtain statistically significant motif matches (compared to a zero-order background model, *P*-value < 0.0001) to *cis*-regulatory promoter regions in five salmonid species (Table 1). Similarly to other studies (e.g. [36, 37]), we defined *cis*-regulatory regions to extend from 1000 bps upstream to 200 bps downstream of transcription start sites of protein-coding genes, thus including the entire untranslated region (UTR) of most genes (Additional file 2: Figure S2). When several motifs matched identical genomic locations (i.e. same start and stop position), we only kept the motif with the lowest *p*-value. Mapping statistics were comparable across salmonid species, with each gene on average harboring from 311 to 439 different non-redundant motif matches in its promoter. Each non-redundant motif matched the promoter of 6062 to 8255 genes on average (corresponding to 10–14% of the genes in these species), with an average of ~ 1.3 matches per gene. Although the Atlantic salmon genome harbors a considerably higher absolute number of motif matches than other species, the per gene count is comparable to the other salmonids. For all species, motif matches are clearly enriched around the transcription start site in what is generally referred to as the core promoter (Fig. 2), which is consistent with observations in other species [37–39].

**Table 1:** Summary of motif matches in SalMotifDB by species. Motifs were mapped to promoter regions spanning − 1000/+ 200 bps up−/down-stream of transcription start sites (FIMO *P*-value < 0.0001). Numbers are given for all motifs as well as for central motifs (non-redundant) and are furthermore divided into numbers were all matches to a promoter is counted (Motif matches) and numbers were only one match per promoter is counted (Gene matches)

		All motifs		Central motifs	
Species	Genes	Motif matches	Gene matches	Motif matches	Gene matches
Arctic char (*Salvelinus alpinus*)	42,439	83,048,193	65,471,635	23,860,740	18,622,927
Atlantic salmon (*Salmo salar*)	81,586	118,076,727	89,320,019	33,945,569	25,359,764
Chinook salmon (*Oncorhynchus tshawytscha*)	48,724	92,515,215	71,056,812	26,665,761	20,186,660
Coho salmon (*Oncorhynchus kisutch*)	46,109	88,330,829	68,692,831	25,395,939	19,551,420
Rainbow trout (*Oncorhynchus mykiss*)	55,685	88,139,627	70,459,150	25,150,603	20,018,446

In addition to raw motif matches, SalMotifDB also allows the user to filter out motif matches that are in repeat regions or in unconserved regions. A repeat library database was built for each salmonid species using RepeatModeler v4.0.3 (http://www.repeatmasker.org) and the genomes were repeat masked using RepeatMasker v4.0.3 (http://www.repeatmasker.org). Genome wide multi-species homeologous block alignments across all the species in the database were generated using Mugsy [40] with Atlantic salmon genome as the reference. These alignments were then used by phastCons [41] to calculate a conservation scores and the most conserved elements in each salmonid species.

### The transcription factors

We extracted the amino acid sequence for all 19,845 metazoa TFs associated to a motif in the motif databases and performed BLAST searches against each salmonid species (NCBI blast+ with *evalue = 0.0001* and *max_target_seqs = 5*). The TFs and their hits to salmonid proteins were then both blasted against the NCBI Conserved Domain Database (CDD) (delta-blast *evalue = 0.0001*) [42], and protein domain similarity was computed using the *Jaccard index.* Salmonid genes with both a significant BLAST hit and a CDD Jaccard index ≥ 0.8 to a TF were considered putative salmonid TFs (Table 2). Considering that the number of genes vary substantially in these species (Table 1), the number of predicted TFs varied much less ranging from 2008 in Arctic char to 2194 in Chinook salmon.

**Table 2:** Summary of TF prediction in the salmonids. The table shows the number of predicted TFs in each salmonid species. The second column contains the number of salmonid genes with significant BLAST hits to TFs with associated motifs in the motif databases. The last column contains the number of salmonid genes with both significant BLAST hits to TFs and a CDD Jaccard index ≥ 0.8

Species	No. genes predicted to be TFs	
	BLAST	BLAST + CDD
Arctic char	2480	2008
Atlantic salmon	2430	2045
Chinook salmon	2737	2194
Coho salmon	2512	2035
Rainbow trout	2761	2235

### The putative regulatory networks

**Fig. 3:**
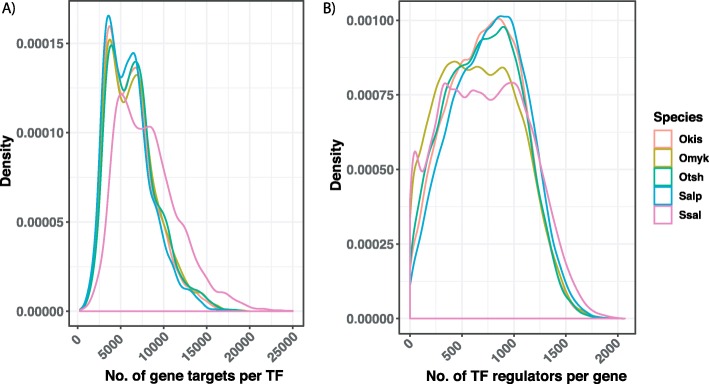
Salmonid-specific putative regulatory networks. **a** The distribution of the number of genes predicted to be regulated by a TF in each species (out-degree). Note that the Atlantic salmon (Ssal) genome has a considerably higher gene count than the other species. **b** The distribution of the number of TFs predicted to regulate a gene (in-degree)

Through motif matching and TF prediction, we have effectively lifted over information on TF-binding site interactions from multi-species databases to salmonid species. By assuming that a TF regulates a gene if one of its associated motifs match in the promoter region of that gene, we have inferred putative global regulatory networks for the salmonid species. These directed networks are highly interconnected with each TF predicted to regulate on average ~ 6000 genes (network out-degree) and each gene predicted to be regulated by on average ~ 700 TFs (in-degree) (Fig. 3). Interestingly, these average numbers hide bimodal-like distributions (i.e. distributions with two peaks). For example, TFs belong to two groups with very different numbers of target genes (Fig. 3a), where the most highly connected group includes a long tail of extremely highly connected TFs with up to 25,000 targets (hubs). It should be noted, however, that these dense networks represent a potential for regulation, and can be made more realistic to a specific cellular context by integrating dynamic data such as expression data or open chromatin data from specific cell types, stress conditions or developmental processes.

### The implementation

The backend of SalMotifDB consists of a MySQL database (database schema available in Additional file 3: Figure S3) and R scripts. The database schema and integrity is managed by the Django web framework. The frontend of SalMotifDB is hosted on an R shiny server that provides a user friendly interface for retrieving data from the database and performing different motif analysis. DNA binding site information such as motif logos, PSSMs and literature references is available through links to footprintDB (http://floresta.eead.csic.es/footprintdb) [29] for open source databases and geneXplain (http://genexplain.com/transfac/) for TRANSFAC© [32]. SalMotifDB is also accessible through an R packages. The R shiny web interface and R package code are publicly available in a GitLab repository accessible from the SalMotifDB web site (https://salmobase.org/apps/SalMotifDB).

## Utility and discussion

### The SalMotifDB web site and R package

**Fig. 4:**
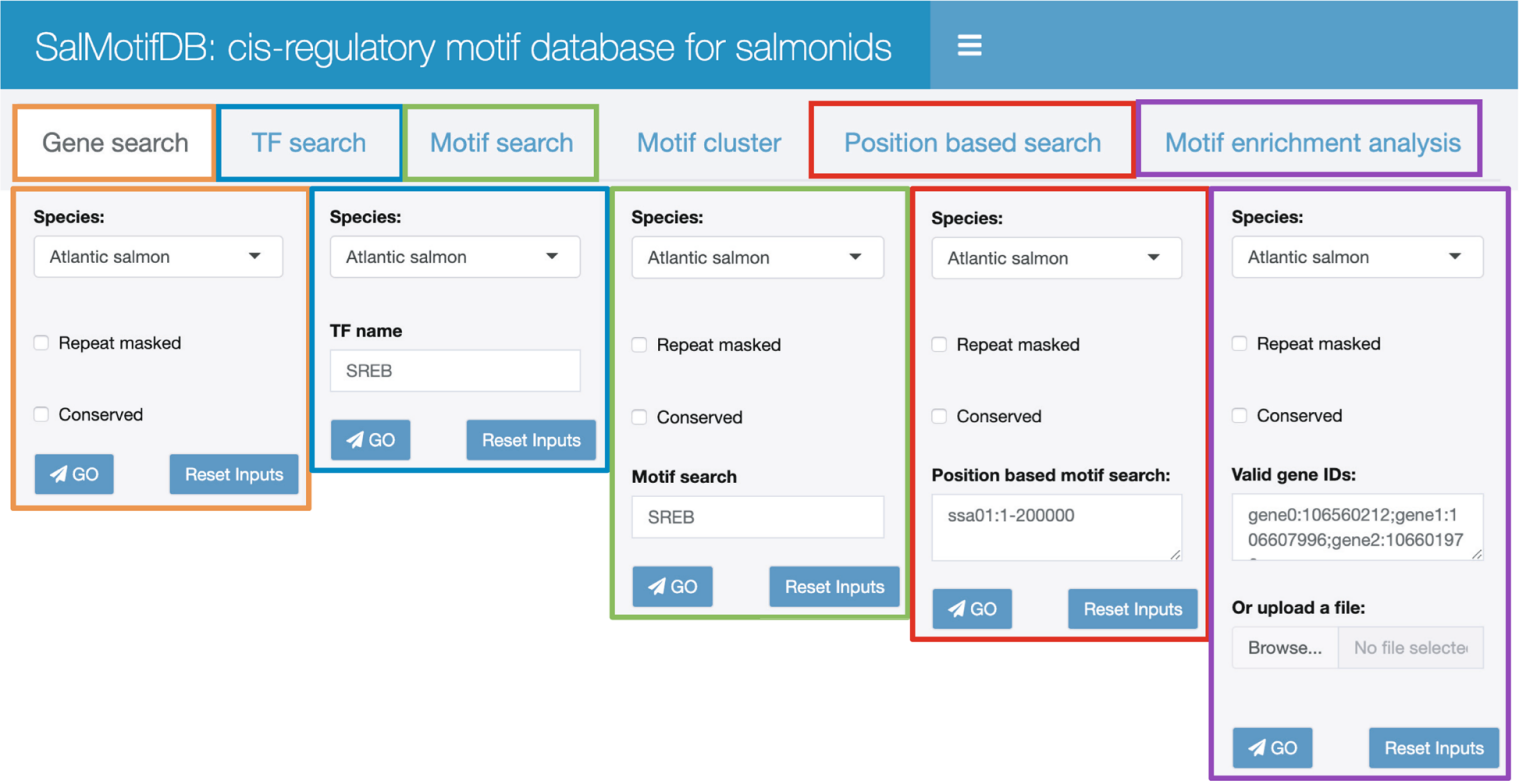
SalMotifDB web interface: menus. The tailored menus for five tools available in SalMotifDB are shown, with example input values included. The Motif cluster tool does contain any tool-specific input

We have implemented an R shiny web interface and an R package (https://salmobase.org/apps/SalMotifDB/) that provides access to the underlying SalMotifDB database through six tools (Fig. 4):

#### Gene search

The gene search tool allows the user to retrieve the motifs that match the promoter region of a query gene. Results include basic information about the gene, individual motif matches with distance from transcription start site, *p*-value and links to the external motif database as well as a graphical representation of where in the promoter the motifs match.

#### TF search

The TF search tool allows the user to search with a TF name and retrieve its motifs from external databases as well as predicted salmonid orthologs with information on BLAST E-value score and shared CDD domains.

#### Motif search

The motif search tool allows the user to search with a motif name, and retrieve details about all matches of that motif to salmonid promoters. Results also include a graphical representation of the motif’s similarity to other motifs.

#### Motif cluster

The motif cluster tool allows the user to explore clusters of similar motifs, and includes graphical representations of the number of motifs from each external database and the similarity structure of the motifs in the cluster. Results also include sequence logos for individual motifs.

#### Position based search

The position based search tool allows the user to specify a genomic region of interest and retrieve details about all motif matches to promoters of genes located in that region.

#### Motif enrichment analysis

The enrichment tool allows the user to input a list of genes (e.g. differentially expressed genes) and identify motifs that match the promoters of these genes more often than expected by chance. The tool gives details about enrichment *p*-values (using the hypergeometric distribution), as well as details about all individual motif matches to promoters of genes in the list. Results also include a visual representation of the regulatory network inferred from the motif enrichment analysis.

Each tool comes with a customized walkthrough explaining the input and output of that tool.

### Examples of utility: lipid metabolism and duplicate divergence

**Fig. 5:**
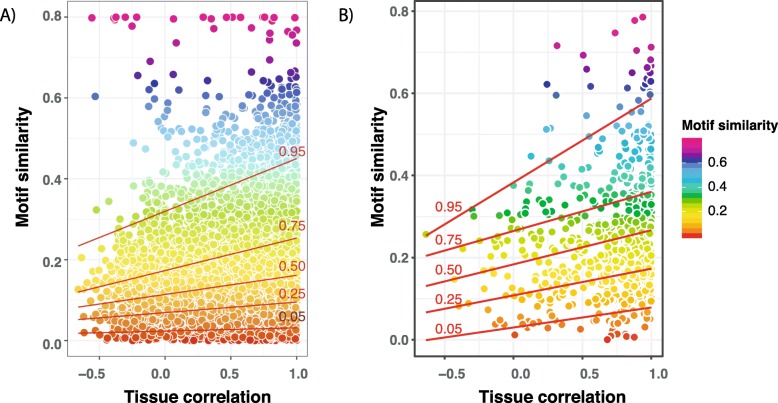
Ohnolog motif and expression similarity. Motif similarity (Jaccard index, y-axis) plotted against tissue expression correlation (Pearson correlation computed over 13 tissues) for ohnolog pairs. Quantile regression line are shown at 0.05, 0.25, 0.5, 0.75, and 0.95. (A) All 10,515 ohnolog pairs and (B) 735 ohnolog TF pairs

We provide two examples of the utility of the SalMotifDB by testing the tool on two sets of genes. Gene set 1 contains genes that are differently regulated between Atlantic salmon that were given feed with contrasting lipid and fatty acid content in fresh water (67 genes with *p* < 0.05 from supplementary Table 8 in ref. [12]). Transcriptional regulation of hepatic lipid metabolism is extensively studied [43] and are known to be conserved across vertebrates, including salmon [44]. This gene set is thus expected to be enriched in motifs associated with the lipid metabolism regulatory network(s) in liver [10, 12]. Indeed, a test for enriched motifs in SalMotifDB showed that promoters in gene set 1 were significantly enriched (*p* < 0.05) for motifs bound by key lipid metabolism regulators such as SREBP-1, PPAR, NF-Y, and SP (Additional file 5: Table S1). Next we tested the tool on a gene set of duplicated gene pairs (ohnologs) originating from the salmonid specific whole genome duplication 80–100 million years ago. Salmonids are used as a model system to understand consequences of whole genome duplication on genome regulatory evolution [2, 13] and we know that about 60% of the retained duplicates display diverged tissue expression profiles [2]. Using SalMotifDB to identify motifs in promoters of duplicated genes in Atlantic salmon (identified using the same approach as in ref. [2]), we then tested the hypothesis that divergence in tissue expression is linked to divergence of the cis-regulatory landscape between gene duplicates. We observed a significant correlation of 0.20 (*p* < 2.2e-16 using Pearson Correlation Coefficient test and *p* = 0.0 using randomization, Additional file 4: Figure S4) between motif similarity (Jaccard index) and tissue expression correlation (Pearson Correlation Coefficient) for 10,515 ohnologs and a correlation of 0.21 (*p* < 2.2e-16) for 735 TF ohnologs (Fig. 5). Furthermore, the data included in SalMotifDB was also recently used to identify associations between groups of duplicated genes displaying similar regulatory evolutionary fates and their promoter motif divergence [14]. Taken together, these analyses demonstrates the utility of SalMotifDB as a tool to improve interpretations and support biological validity of gene expression analyses and help understand the mechanistic drivers of gene regulation evolution.

### Future

SalMotifDB relies on transcription factor - motif interactions observed in different species, tissues and experimental conditions. Furthermore, we only consider matches of these motifs to relatively restricted upstream regions in our selected salmonid species. Despite these limitations, we here show the utility of this motif database in identifying biologically sound links between *cis*-regulatory landscapes and gene expression patterns in salmon. Future developments of this database include adding genome tracks for epigenetic profiling such as ATAC-Seq. This will greatly improve prediction of TF binding in celltype-, environment-, and developmental-stage-dependent contexts, as well as extending the use of the motif database to distal regulatory regions such as enhancers.

## Conclusion

We show that the SalMotifDB is an effective tool for extracting information about transcription factor binding sites, transcription factors, and gene regulatory networks in salmonid species. This database is an important resource for future studies that aims to gain mechanistic understanding of regulation of transcription, and thereby salmonid evolution and physiology.

## Additional files

Additional file 1: **Figure S1.** A radial tree displaying the similarity structure of a selected motif cluster. The inner node represent the cluster name (cluster-393), nodes in the second layer represent the motif databases, the third layer represent motif/TF names and the outer layer represent consensus motifs (not available for TRANSFAC). The central motifs of each motif database cluster are highlighted in red. (PDF 260 kb)

Additional file 2: **Figure S2.** Density plot of the lengths of the untranslated regions (UTRs) of genes in the salmonid genomes. (PDF 260 kb) (PDF 17 kb)

Additional file 3: **Figure S3.** SalMotifDB database schema for Atlantic salmon. (PDF 156 kb)

Additional file 4: **Figure S4.** Motif similarity (Jaccard index, y-axis) plotted against tissue expression correlation (Pearson correlation computed over 13 tissues) for 10,515 randomly selected ohnolog pairs. Quantile regression line are shown at 0.05, 0.25, 0.5, 0.75, and 0.95 and, in contrast to actual ohnologs, indicate no correlation. (PNG 207 kb)

Additional file 5: **Table S1.** Motifs in SalMotifDB that were significantly enriched (*p* < 0.05) in the promoters of genes in gene set 1: genes that are differently regulated between Atlantic salmon that were given feed with contrasting lipid and fatty acid content in fresh water. (XLSX 35 kb)

## Data Availability

All data analysed in this study are publicly available through provided references. The code for the web tool and R package, as well as database content, can be downloaded at https://salmobase.org/apps/SalMotifDB.

## Abbreviations

ATAC-Seq: Assay for Transposase-Accessible Chromatin using Sequencing
BLAST: Basic Local Alignment Search Tool
CDD: Conserved Domain Database
CIGENE: Centre for Integrative GENEtics
CIS-BP: Catalog of Inferred Sequence Binding Preferences
DNA: DeoxyriboNucleic Acid
FIMO: Finding Individual Motif Occurrence
HOCOMOCO: HOmo sapiens COmprehensive MOdel COllection
HT-SELEX: High-Throughput Systematic Evolution of Ligands by EXponential enrichment
MySQL: My Structured Query Language
NCBI: National Center for Biotechnology Information
NF-Y: Nuclear transcription Factor Y
PPAR: Peroxisome Proliferator-Activated Receptors
PSSM: Position Specific Scoring Matrices
RSAT: Regulatory Sequence Analysis Tool
SalMotifDB: Salmonid Motif DataBase
SMiLE-Seq: Selective Microfluidics-based Ligand Enrichment followed by Sequencing
SP: Specificity Protein
SREBP: Sterol Regulatory Element Binding Protein
TF: Transcription Factor
TRANSFAC: TRANScription FACtor database
UniPROBE: Universal PBM Resource for Oligonucleotide-Binding Evaluation
UTR: UnTranslated Region
