# Supplementary figures and images for: SalMotifDB: a tool for analyzing putative transcription factor binding sites in salmonid genomes
Source: BMC Genomics. 2019 Sep 2;20:694. doi: 10.1186/s12864-019-6051-0 (PMC6720087; doi:10.1186/s12864-019-6051-0)

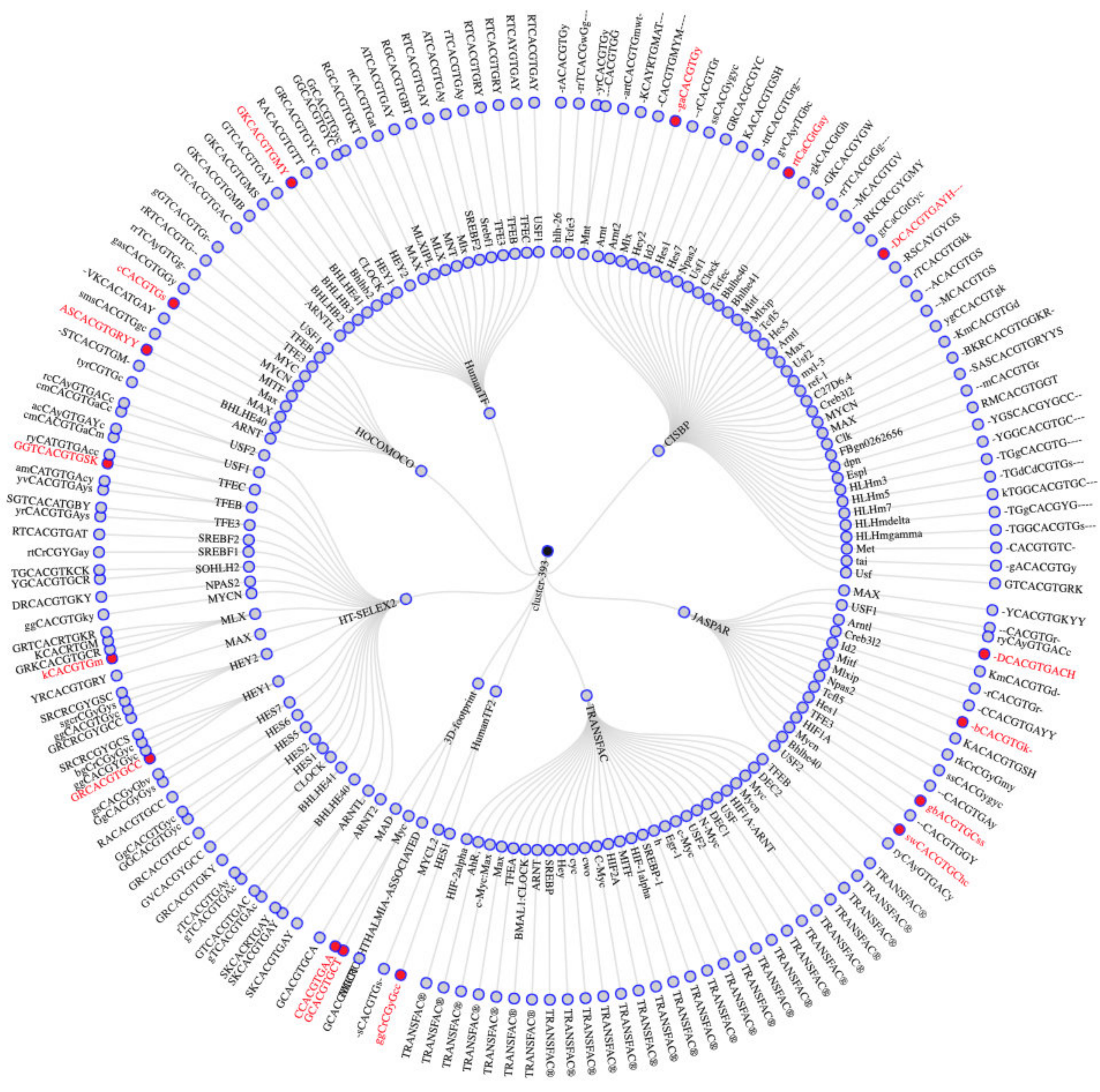

Supplement: Supplementary file 1 — Figure S1. A radial tree displaying the similarity structure of a selected motif cluster. The inner node represent the cluster name (cluster-393), nodes in the second layer represent the motif databases, the third layer represent motif/TF names and the outer layer represent consensus motifs (not available for TRANSFAC). The central motifs of each motif database cluster are highlighted in red. (PDF 260 kb) [file 12864_2019_6051_MOESM1_ESM.pdf]

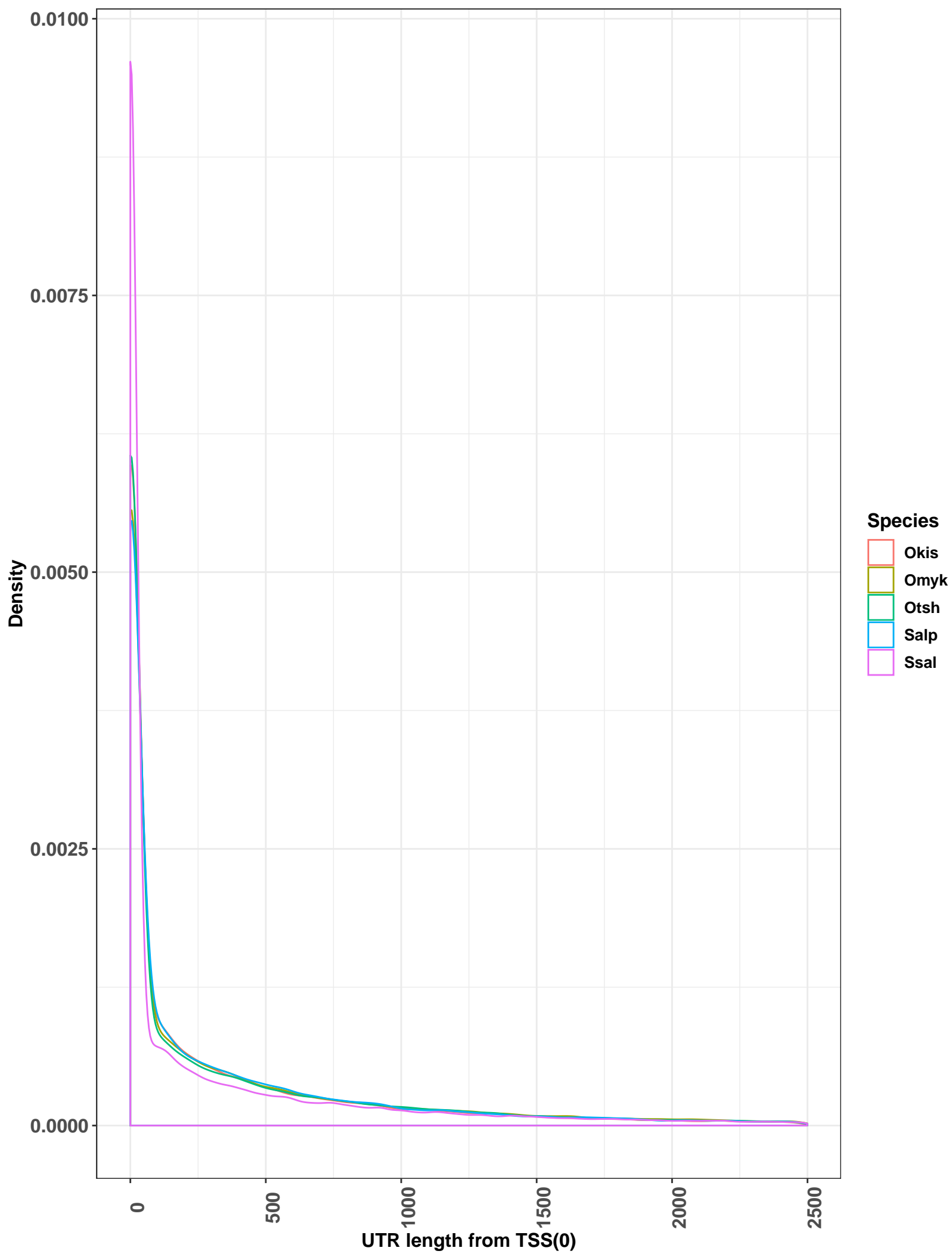

Supplement: Supplementary file 2 — Figure S2. Density plot of the lengths of the untranslated regions (UTRs) of genes in the salmonid genomes. (PDF 260 kb) (PDF 17 kb) [file 12864_2019_6051_MOESM2_ESM.pdf]

## SalMotifDB database schema for Atlantic salmon

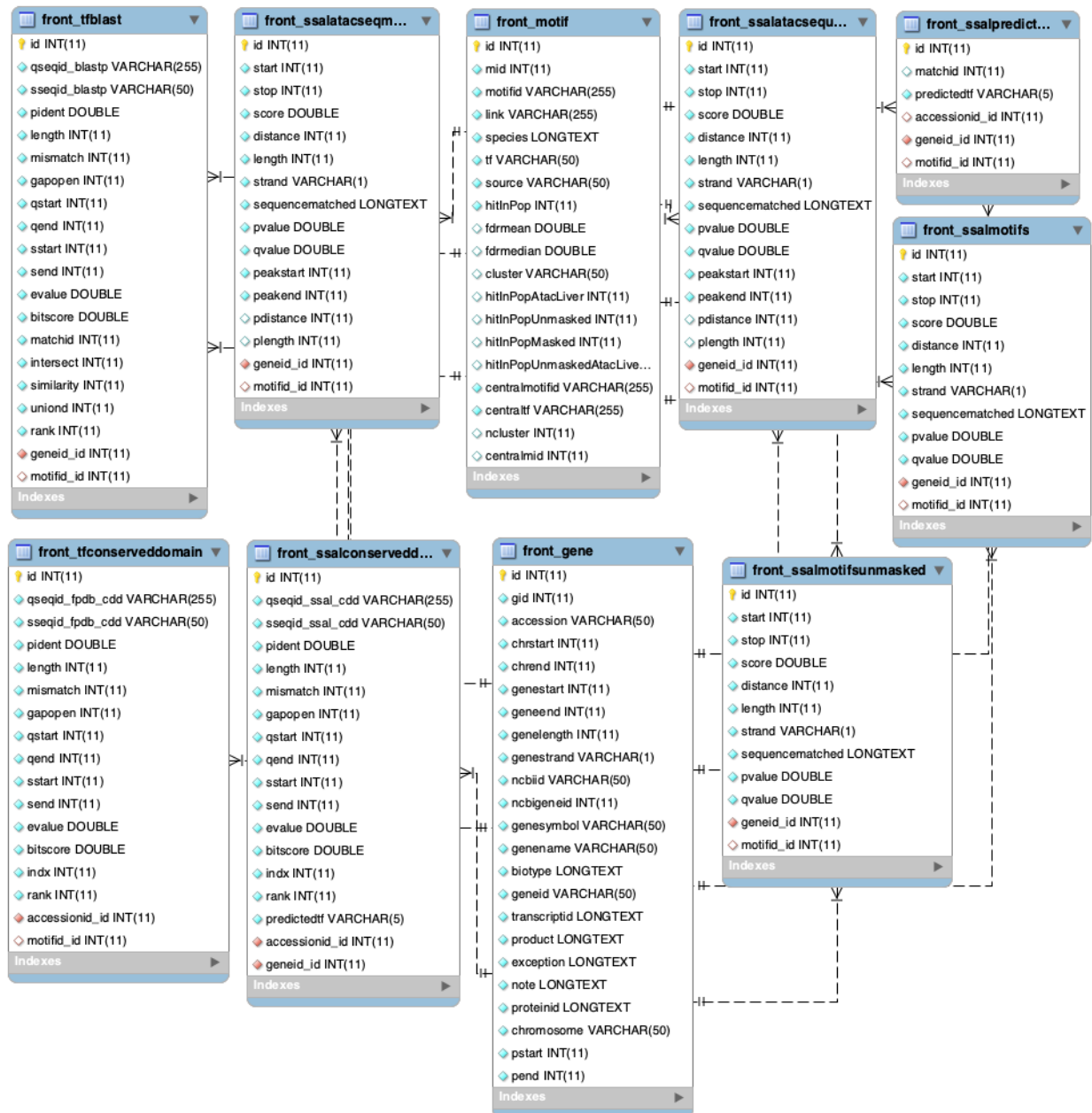

Supplement: Supplementary file 3 — Figure S3. SalMotifDB database schema for Atlantic salmon. (PDF 156 kb) [file 12864_2019_6051_MOESM3_ESM.pdf]

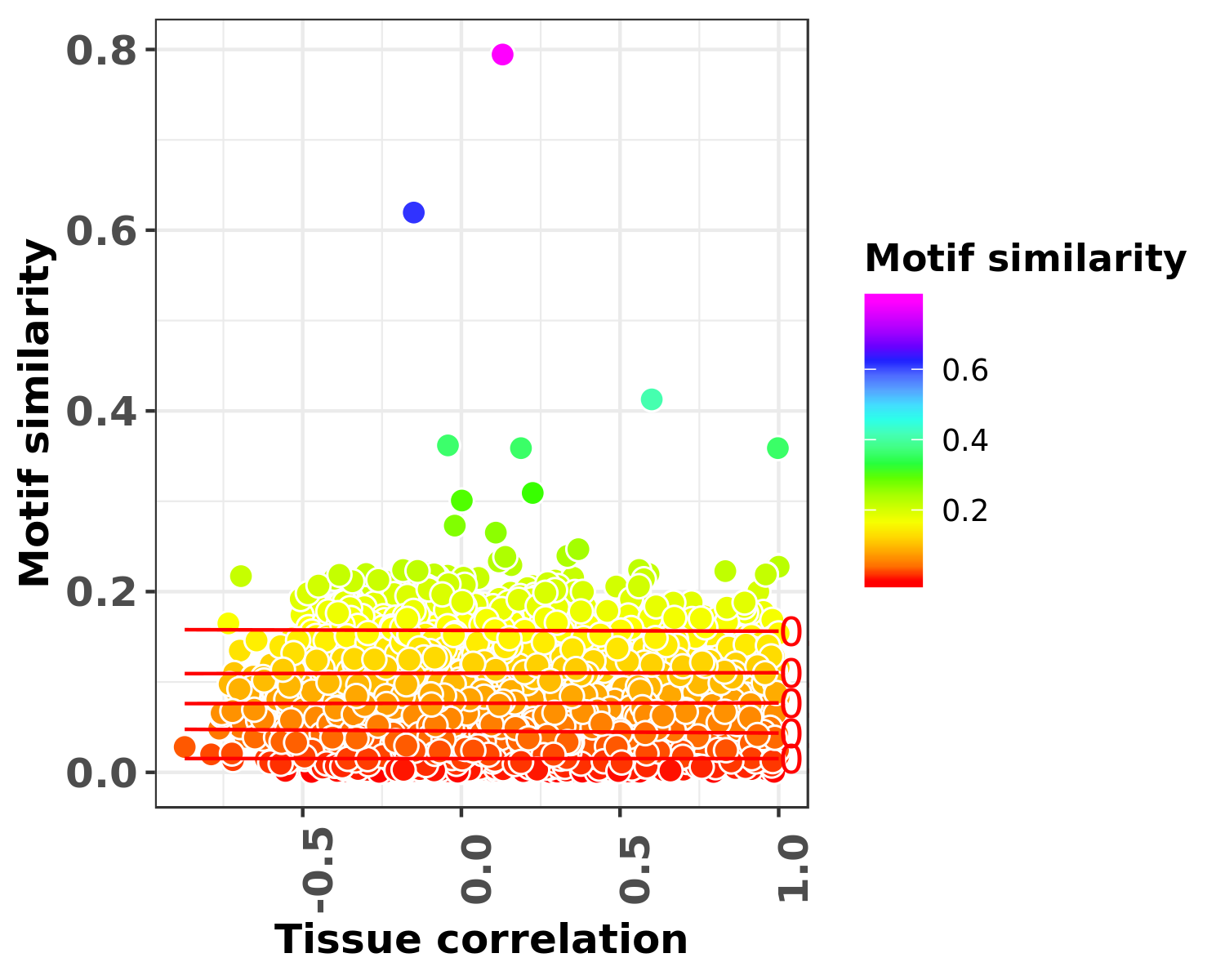

Supplement: Supplementary file 4 — Figure S4. Motif similarity (Jaccard index, y-axis) plotted against tissue expression correlation (Pearson correlation computed over 13 tissues) for 10,515 randomly selected ohnolog pairs. Quantile regression line are shown at 0.05, 0.25, 0.5, 0.75, and 0.95 and, in contrast to actual ohnologs, indicate no correlation. (PNG 207 kb) [file 12864_2019_6051_MOESM4_ESM.png]
